# The Spread of Typhoid Fever in London by the Milk-Supply

**Published:** 1873-10-15

**Authors:** 


### Medical Examiner.

*A Semi-Monthly Journal of Medical Sciences.*

EDITED BY

N. S. DAVIS, M. D., AND F. H. DAVIS, M. D.

Chicago, Oct. 15th, 1873.

### EDITORIAL.

### THE SPREAD OF TYPHOID FEVER IN LONDON BY THE MILK-SUPPLY.

The *British Medical Journal*, of Sept. 6th, contains an extended account of the recent alarming outbreak of Typhoid Fever in London, and the seeming connection which has been traced between its spread and the supply of milk from a certain dairy.

According to an editorial in the *Philadelphia Medical Times*, Dr. Murchison, lately in charge of the Fever Hospital, and famous the world over for his work on Continued Fever, was the first to suspect the milk-supply as a source of contagion.

"Several cases occurred in the household of Dr. Murchison. Feeling confidence in the sanitary arrangements of his dwelling, he was led to suspect some extramural cause, and instituted inquiries among several of his medical friends in the neighborhood, in whose families there were cases of typhoid fever. He found that in every instance the milk-supply, which he already regarded as the possible source of infection, was the same as that of his own household. He at once communicated with the medical officer of the parish; the milk company were summoned to suspend the sale of milk from their dairy, and a commission of inquiry appointed. It was found that of the eight farms with which the company contracted for the supply of milk, seven presented no probability of enteric contamination, but that to the eighth the most suspicious circumstances attached. The farmer, a strong middle-aged man, believed by his medical attendant to be suffering from a "fatty heart," was taken ill with indefinite symptoms about the second week in May. A fortnight later a consulting physician declared his case to be one of typhoid fever. His opinion was, however, disregarded, and the man was allowed to remain out of bed and walk about whenever he felt able to do so. For the first few days in June he had a copious diarrhœa, with putrid and bloody discharges; this was followed by constipation, for which a dose of castor oil was given.

"On June 8, as he was being lifted from his bed, he died. On account of the suddenness of his death, the doubt about the original diagnosis, and the fact that he seemed to have recovered from his recent illness, his medical attendant entered as the cause of death "heart-disease;" but fatal collapse is certainly of not unfrequent occurrence in typhoid fever, and the circumstance rather confirms than discredits that diagnosis.

"The only privy on the premises was at the upper part of the yard, the bottom of its pit being two or three feet above the level of the well from which drinking-water was obtained, and the ground affording opportunity for free drainage.

"At the time of the investigation the son of the farmer had just been taken sick with typhoid fever. It was also found that there had been an epidemic of the same disease in some neighboring villages.

"It seems that the milk company, being a corporation, and therefore soulless, objected very strongly to their business being interrupted, asking for proof that the typhoid-fever poison was in the milk, although they had been assured that out of the thirty-seven families stricken with the fever, thirty-five were supplied with their milk. They wanted, forsooth, this mysterious entity—the typh-poison—this thing that eye hath not seen, precipitated from their milk, weighed, analyzed, and delivered in sealed packages at their office, so that they might put it into the hands of a chemist to have its identity proven!

"Even the medical health officer of the parish tried to interfere, but to no avail; and finally the sale of the poison was only checked after some days of discussion, when apparently public opinion and the fear of future damages to be judicially awarded induced this most worthy body corporate to investigate the matter.

"The following extract from a letter of Dr. Murchison will show the evidence relied on by him and Dr. Jenner as demonstrating the origin of the disease:

"'Out of sixty families (placed under the same conditions as to drainage, etc., as their neighbors) of the better class, already known to us, in which enteric fever has appeared within the last month, all but one have used your milk.

"'In certain families not supplied by your company, individuals have been attacked with enteric fever who have accidentally partaken of your milk; and then these individuals have been the only members of the family who have suffered.

"'An unusually large proportion of the patients have been young children who consume much milk. In certain families, children who drank water, but not milk from your dairy, have remained exempt; while the children who drank Dairy Reform milk have suffered; and of the adults who have taken the fever, most have been milk-drinkers.'"

